# Comparison of clinical examination with orbital MRI evaluation in patients with thyroid orbitopathy - do we need imaging and when?

**DOI:** 10.1186/1756-6614-6-S2-A34

**Published:** 2013-04-05

**Authors:** Aleksandra Król, Jolanta Krajewska, Barbara Michalik, Ewa Paliczka-Cieślik, Michał Kalemba, Beata Jurecka-Lubieniecka, Kornelia Hasse-Lazar, Sylwia Szpak-Ulczok, Łukasz Zarudzki, Barbara Jarząb

**Affiliations:** 1 Department of Nuclear Medicine and Endocrine Oncology, Maria Sklodowska Curie Memorial Cancer Centre and Institute of Oncology, Gliwice Branch, Gliwice, Poland

## Introduction

The frequency of Graves’ orbitopathy (GO) in patients with Graves’ disease (GD) depends on the method of assessment. Diagnosis based on clinical symptoms is made in about 30-50% patients, more often (70%) after ophtalmological examination with evaluation of intraocular pressure, whereas MRI reveals GO in up to 90%. Proper identification of active phase of the disease is crucial.

## Aim of the study

The aim of the study was to assess the concordance between clinical examination performed by endocrinologist and MRI GO diagnosis, especially to evaluate the sensitivity of both methods in identification of active phase of the disease.

## Material and methods

MRI of the orbits was performed in 85 hyperthyroid GD patients qualified to 131I therapy. The clinical GO evaluation based on CAS and NOSPECS scores was done in all subjects, however no information concerning MRI result was provided to the physician.

## Results

Radiological signs of GO were present in 78/85 patients (92%). Active phase was diagnosed in 20/85 subjects, among them in 16/20 on the basis of clinical evaluation and in 15/20 by MRI. Concordant diagnosis was made in 11 cases, whereas in the remaining 9 subjects active phase of GO was recognized by clinical (5 pts) or radiological (4 pts) features only. 7 patients with no changes in MRI were also negative in clinical examination - 100% accurate results. In 63 patients inactive/chronic GO was described in MRI, whereas in clinical examination active disease was stated in 5/63 cases, mild GO in 19/63 and no symptoms of GO in 39/63.

## Conclusions

Clinical evaluation based on CAS score shows high sensitivity in detecting active GO. MRI is recommended in doubtful cases.

